# The Effects and Measures of Auricular Acupressure and Interactive Multimedia for Smoking Cessation in College Students

**DOI:** 10.1155/2014/898431

**Published:** 2014-06-02

**Authors:** Mei-Ling Yeh, Pei-Lan Wang, Jaung-Geng Lin, Mei-Ling Chung

**Affiliations:** ^1^School of Nursing, National Taipei University of Nursing and Health Sciences, 365, Ming-Te Road, Pei-Tou, Taipei 112, Taiwan; ^2^Department of Nursing, Hsin Sheng College of Medical Care and Management, Taoyuan, Taiwan; ^3^Graduate Institute of Acupuncture Science, China Medical University & China Medical University Hospital, Taichung, Taiwan; ^4^School of Chinese Medicine and Acupuncture Science, China Medical University, 91 Hsueh-Shih Road, Taichung 40402, Taiwan

## Abstract

The earlier one starts to smoke, the more likely it is that one's tobacco use will increase. Either auricular acupressure or multimedia education could improve physiological health status and reduce smoking for young smokers. This study aimed to evaluate the effects of a 10-week auricular acupressure (AA) and interactive multimedia (IM) on smoking cessation in college smokers. A pre- and posttest control research design with two experiments (AA and IM) and one control was used. Thirty-two participants were in each of three groups. A significant difference from pretest to posttest among three groups was exhibited on carbon monoxide (CO), cotinine, and nicotine dependence. Scheffe's * post hoc* test found significances on CO in the AA between the IM and the control and cotinine and nicotine dependence between the AA and the control. After controlling the covariates, the main effect of the group was no difference in all outcomes. The interventions, especially AA, may contribute to a decrease of CO, cotinine, and nicotine dependence along with the time change. An analysis without controlling influences may overestimate interventional effects.

## 1. Introduction

Young adulthood is a critical period during which nicotine dependence and smoking behavior develop. About 21.5% of young adults become daily smokers [1]. Failure to stop smoking may result in increased smoking intensity and cumulative cigarette consumption [2], increased susceptibility to autoimmune diseases [3], higher risk of metabolic disorders [4], and early decline in lung function [2]. Forced expiratory volume in the first second (FEV1) is an early indicator of air flow limitation in asymptomatic smokers. Moderate to heavy smokers have a 15 ml/year larger decline in FEV1 than in nonsmokers, and when decline was expressed as FEV1 percent predicted (FEV1%), there is no gender difference [5]. FEV1 can recover after smoking cessation [2]. Previous studies, in addition to suggesting the measure of pulmonary function [6], found that carbon monoxide (CO), cotinine, and nicotine dependence would be normalized [7, 8].

According to the transtheoretical model of health behavior change [9], people in any stage of precontemplation, contemplation, preparation, and action maintain present readiness for changing smoking motivation and behavior. Smoking cessation intervention would help move upward the smokers' stages of change that were positively influenced by one's self-efficacy [10, 11]. Moreover several factors influence the decision of young smokers to quit, for instance, years of smoking [6, 8], peer smoking [7, 12], and parental smoking [13–15]. The earlier one starts to smoke, the more likely it is that one's tobacco use will increase.

Auricular acupressure (AA) is a simple, noninvasive intervention that can decrease nicotine dependence and improve the refusal smoking self-efficacy [13, 16]. Acupoint stimulation can increase endorphin [17] and serotonin [18] that are transmitted to the brain and particular organs in the rest of the body through nerves and meridian so as to modulate physiological reactions [19]. AA had positive effects on smoking cessation as measured by CO or cotinine [8, 20] but not in the others [21]. The systematic review concluded that no particular acupuncture is superior to any other [22]. However, the poor sensitivity of CO and cotinine levels as indicators of smoking activity has been reported in young smokers [23]. Outcome measures of AA in young smokers need further studies.

The flexible, convenient, and interesting content of interactive multimedia (IM) may help smokers accelerate the process of behavior change [24], improving positive cognition and attitude of smoking cessation [7], reinforcing self-efficacy to resist smoking [7], and contributing to a decreased nicotine dependence [25]. However, the effectiveness of IM in cessation remains controversial because of measurement issues, such as lack of biochemical verification [26].

## 2. Purpose 

This study aimed to evaluate the main effects of AA and IM on smoking cessation in college smokers. Significant differences in FEV1%, exhaled CO, serum cotinine, and nicotine dependence were hypothesized among the groups.

## 3. Methods

### 3.1. Research Design and Participants

This study used a pre- and posttest control research design with two experimental groups (one given AA and the other IM for 10 weeks) and one control group (receiving no intervention for 10 weeks). Participants were recruited on a voluntary basis from one university in the north of Taiwan and were nonrandomly assigned to one of three groups based on their preference. Inclusion criteria were enrolled in the study university: over 18 years, CO > 6 ppm, serum cotinine >100 ng/mL, no abnormally shaped cutaneous lesions at the application sites, and not treated for smoking. Based on FEV1% with a medium effect size (*f* = 0.3) and 80% power at 5% level of significance using repeated-measure 3 times, a total of 75 subjects would be minimally required. With an estimated loss of 20%, 90 subjects were necessary.

### 3.2. Interventions

AA is a smoking cessation method involving pressure applied to six common auricular acupoints of* shenmen, lung, stomach, mouth, subcortex,* and* hunger* [6–8, 13]. The stimulation of the* shenmen* can raise endorphin and thus decrease withdrawal syndromes [19] and nicotine dependence [27]. Stimulation of the* lung, stomach, mouth*, and* subcortex* can normalize qi energy flow and harmonize the stomach and heart [28]. Stimulation of the* hunger* can prolong gastric motility and decrease appetite [19]. A seed-embedding method was used to prolong stimulation at the selected acupoints. An adhesive patch containing the seed was replaced weekly. Participants were instructed to press each auricular acupoint for at least 1 minute per time and 3–5 times per day for 10 weeks. AA was given by a researcher who had earned 40 traditional Chinese medicine related formal credits and was validated by two physicians who practiced traditional Chinese medicine.

The format of IM contained files of text, animation, graphics, picture, sound, and image to provide instructions. The major contents of the program CD included the impact of smoking on health and the environment, information about smoking hazards, strategies for smoking cessation, and problems arising during the smoking cessation period and their solutions [7, 13]. The participants in the computer lab were given the instruction through IM for at least 20 min per time and once a week for 10 weeks. Instead of playing the 20 min whole video, the version of IM is capable of playing only the selected segments, which is designed to facilitate practice and review. Therefore, the participants could watch it without time limits and effectively select the part as needed.

### 3.3. Ethical Considerations

The ethical approval was obtained from the study university. Subjects who met the inclusion criteria were invited to participate. Informed consent was then obtained from all participants and assurance was given to them that the data would be handled anonymously. After completing the pretest of outcome measures, on the schedule the intervention was given. Participants were also provided verbal information concerning care of the ear seed. A posttest was conducted at the end of the intervention.

### 3.4. Outcome Measures

FEV1 is the amount of air which can be forcibly exhaled from the lungs in the first second of a forced expiratory maneuver [5]. Forced vital capacity (FVC) is the amount of air which can be forcibly and maximally exhaled out of the lungs after a maximal inhalation. FEV1% is the ratio of FEV1 to FVC, with 75–80% in healthy adults. FEV1 and FVC were assessed lung function using a Spirolab-II spirometer (Medical International Research, Rome, Italy). Exhaled CO and serum cotinine are objective biomarkers to distinguish smokers from nonsmokers [29]. Exhaled CO was measured by a Micro Medical meter (Micro Co., Rochester, Kent, England), with a cutoff value of under 6 ppm used to identify nonsmokers [30]. Cotinine was determined by enzyme-linked immunosorbent assay (ELISA), the cotinine direct ELISA kit (Immunalysis Co., Pomona, CA), and an ELISA reader (Molecular Devices Co., Menlo Park, CA). Cotinine under 100 ng/mL indicated the absence of tobacco use [7].

The demographic characteristics involved age, age of initial smoking, age of regular smoking, times tried to quit smoking, peer smoking, family smoking, second-hand smoking, and parental education levels. Severity of nicotine dependence was measured using the FTND that has satisfactory validity and reliability [31]. The higher likelihood of nicotine dependence was indicated by higher score. A modified refusal smoking self-efficacy scale was used to measure the resistance to smoking in different circumstances [32] and has satisfactory validity and reliability [7]. A higher score indicated a greater likelihood that the individual could control smoking intention and behavior.

### 3.5. Data Analysis

Statistical analyses were performed using the IBM SPSS 20.0. The 5% level of significance was used to confirm. Demographic data was analyzed by the descriptive and univariate analysis. The *χ* ^2^ tests and one-way ANOVA, when applicable, were conducted separately to compare the among-group difference in main outcome variables before (pretest) and after (posttest) the course of the intervention. A general linear mixed model was used fixed effects of intervention, time, and the interaction of intervention and time, and a random subject effect, followed by a Bonferroni test to compare the effects of smoking cessation.

## 4. Results

Of the 112 male participants recruited, 16 withdrew from the study for academic reasons, with the attrition rate of 14.3%. Finally, 96 were in this study, with the mean age of 22.72–23.47 years at the start of the study, 15.13–15.75 years at smoking initiation, and 15.94~16.56 years when smoking became habitual. Of which, 69.79% participants quit smoking for more than 24 hr, with 1.81 ± 0.22 times tried to quit smoking.  Table 1 shows the demographic homogeneity of the three groups.

Table 2 presents the results of FEV1%, CO, cotinine, and nicotine dependence. Before the interventions, the mean FEV1% was 89.66%, with 15 participants having FEV1% of less than 80%. The results of ANOVA showed no significant difference among three groups at pretest on FEV1% (*F* = 0.40, *P* = 0.67), CO (*F* = 0.40, *P* = 0.67), cotinine (*F* = 2.22, *P* = 0.11), and nicotine dependence (*F* = 1.74, *P* = 0.24). A significant difference from pretest to posttest among three groups was exhibited on CO (*F* = 5.41, *P* = 0.01), cotinine (*F* = 4.39, *P* = 0.02), and nicotine dependence (*F* = 10.50, *P* < 0.001) but not in FEV1% (*F* = 0.38, *P* = 0.69). The Scheffe's* post hoc* test found significances on CO in the AA between the IM (*P* = 0.03) and the control (*P* = 0.02), on cotinine between the AA and the control (*P* = 0.02), and nicotine dependence between the AA and the control (*P* < 0.01).

Table 3 presents the results of mixed models of FEV1%, carbon monoxide, cotinine, and nicotine dependence. After controlling the variables of time, father smoking, peer smoking, refusal smoking self-efficacy, and/or smoking years, the result of the general linear mixed model on the main effect of group was not significant difference across time in FEV1%, CO, cotinine, and nicotine dependence (*P* > 0.05). Regardless of interventions, the statistical significance in the main effect of time was significantly different in CO and nicotine dependence (*P* < 0.001). The interaction between group and time was significant in CO (*P* = 0.02) and nicotine dependence (*P* < 0.001). The participants had no adverse effect associated with AA during this study period.

## 5. Discussion

### 5.1. Effects of the Interventions

This study found the effect of the AA and IM interventions on smoking cessation for college smokers in CO, cotinine, and nicotine dependence, but not in FEV1%. This was similar to other studies [8, 21]. However, the interventions reduced smoking behavior but did not lead to normalization of pulmonary function, and this effect differed from one of the other studies [5]. FEV1 should peak in early adulthood; however, this study found a preintervention decline in pulmonary function in the sample of young college smokers. This result is in agreement with other studies [33]. With age, the decline is even more obvious [34].

The interventions, especially AA, could assist college smokers to decrease their CO, cotinine, and nicotine dependence. It seems AA may facilitate progression to more advanced stages of changing smoking behavior. The effect of IM on smoking cessation is slightly supported in this study, which is not entirely in agreement with other studies [8, 35, 36]. Maybe this is because previous studies lacked biochemical verification [26]. In addition, lack of effects may be due to adherence and frequency of use by participants, especially male participants [37]. Therefore, this study suggests that frequency of use and process may be factors influencing the efficacy of intervention. In addition, using IM approaches alone for smoking cessation may not be effective. Some studies support the use of combined rather than single interventions to stop smoking [7, 8, 38]. It is noted that the effect of AA on smoking cessation became not obvious after controlling the variables of time, father smoking, peer smoking, refusal smoking self-efficacy, and/or smoking years in the statistical analysis in this study. As mentioned previously those confounders and the time effect should be considered [6–15]. This result may exhibit the correction for possible overestimation of the effect of AA and IM.

### 5.2. Measurement Issues of Smoking Cessation

Some issues related to the assay of these biomarkers in young smokers have surfaced. First, the specificity of CO and cotinine was high, but their sensitivity was low in this study. The levels of CO and cotinine were below the nonsmoking cutoff value in 6 and 11 participants, respectively, and were both below their cutoff values in 2 participants. This finding is similar to that found in one previous study [29]. The biological half-life of CO is 4-5 hr in sedentary adults. Physical activity and rate of pulmonary ventilation affect CO elimination. The half-life could be as brief as 1 hr during exercise [39]. CO might also become a less sensitive biomarker if smoking behavior was restricted in a nonsmoking environment. In addition, exposure to traffic exhaust and other pollutants resulted in exhaled CO levels of 2–6 ppm [35]. Cotinine has a long biological half-life (15–40 hr) and it would increase and persist when smoking becomes a regular habit [29], which may contribute to a decrease in its sensitivity as a biomarker of change in smoking activity. Second, this study and another study [30] found that CO and cotinine were less correlated although both were sensitive indicators of nicotine dependence [31]. Although these two may be suitable as biochemical markers, other indicators are required.

### 5.3. Study Limitations

The potential threats to validity included selection and treatment assignment bias. The Hawthorne effect was also a threat because participants were aware that they were being observed. Thus, findings should be interpreted with caution because of the limitations of the study design. This study had the effect of smoking cessation during a short period, but a long-standing effect remains unknown. Longitudinal studies in young smokers of various ages, in different genders and in various geographic locales, are recommended. Although this study supports the effect of smoking cessation, this effect was not obvious after adjusting for the covariates. Future studies should clarify the influences of these covariates. Additional studies are needed to further validate the impact of interventions on smoking cessation.

## 6. Conclusion

A review of the literature indicates that this study is the first to examine the effects and the measures of AA and IM on FEV1, CO, cotinine, and nicotine dependence as biomarkers of smoking cessation in college smokers. The interventions, especially AA, may contribute to a decrease of CO, cotinine, and nicotine dependence along with the time change. However, the refusal smoking self-efficacy and smoking years of college smokers, as well as the smoking habits of their fathers and peers are important influences on smoking cessation. An analysis without controlling influences may overestimate interventional effects. Appropriate, even combined, smoking cessation interventions should be considered. Some issues related to the assay of biomarkers have surfaced.

## Figures and Tables

**Table 1:** Demographic characteristics of the three groups.

Variables/groups	AA	IM	Control	*P*
	M ± SD, *n* (%)	M ± SD, *n* (%)	M ± SD, *n* (%)	
Age at study time (yr)	23.47 ± 3.07	23.09 ± 3.12	22.72 ± 3.01	0.62
Age of initial smoking (yr)	15.75 ± 1.57	15.53 ± 1.14	15.13 ± 1.54	0.21
Age of regular tobacco use (yr)	16.56 ± 1.63	15.94 ± 1.05	16.03 ± 1.20	0.13
Times tried to quit smoking	2.16 ± 2.01	1.44 ± 1.85	1.84 ± 2.36	0.40
Quitting smoking over 24 hr				0.45
Yes	25 (78.1)	21 (65.6)	21 (65.6)	
No	7 (21.9)	11 (34.4)	11 (34.4)	
Peer smoking				0.50
Yes	22 (68.8)	26 (81.2)	23 (71.9)	
No	10 (31.2)	6 (18.8)	9 (28.1)	
Family smoking				0.84
Yes	19 (59.4)	21 (65.6)	21 (65.6)	
No	13 (40.6)	11 (34.4)	11 (34.4)	
With second-hand smoking				0.72
Yes	22 (68.8)	20 (62.5)	23 (71.9)	
No	10 (31.2)	12 (37.5)	9 (28.1)	
Father's education level				0.68
Junior high	3 (9.4)	1 (3.1)	3 (9.4)	
Senior high	11 (34.4)	10 (31.3)	12 (37.5)	
Vocational college	12 (37.5)	16 (50)	11 (34.4)	
University and over	6 (18.8)	5 (15.6)	6 (18.7)	

*n* = 32 in each group; AA: auricular acupressure; IM: interactive multimedia.

**Table 2:** FEV1%, carbon monoxide, cotinine, and nicotine dependence at pre- and posttests.

Variables	AA	IM	Control
	M ± SD	M ± SD	M ± SD
FEV1 %			
Pretest	1.04 ± 0.09	1.00 ± 0.10	1.00 ± 0.10
Posttest	1.04 ± 0.09	1.01 ± 0.09	1.02 ± 0.09
Carbon monoxide (ppm)			
Pretest	13.16 ± 5.07	12.81 ± 4.13	12.03 ± 4.00
Posttest	10.31 ± 4.28	11.75 ± 3.55	11.09 ± 3.64
Cotinine (ng/mL)			
Pretest	732.44 ± 814.11	946.25 ± 1074.39	755.91 ± 831.26
Posttest	407.69 ± 496.68	683.19 ± 775.90	756.06 ± 807.32
Nicotine dependence			
Pretest	5.09 ± 2.19	5.59 ± 1.76	6.16 ± 1.71
Posttest	2.53 ± 2.36	4.09 ± 1.89	5.75 ± 1.83

*n* = 32 in each group; AA: auricular acupressure; IM: interactive multimedia; FEV1: forced expiratory volume in the first second.

**Table 3:** The results of mixed models of FEV1%, carbon monoxide, cotinine, and nicotine dependence.

Parameters	FEV1%			Carbon monoxide			Cotinine			Nicotine dependence		
	Beta	SE	*F*	Beta	SE	*F*	Beta	SE	*F*	Beta	SE	*F*
Intercept	1.03	0.02	199.49***	7.78	1.35	47.87***	−84.79	264.26	0.02	32.00	3.51	157.11***
Time	−0.02	0.02	1.36	0.90	0.46	20.66***	−36.58	80.17	1.22	−1.60	1.03	41.11***
Father smoking	−0.07	0.03	6.57*	−1.63	1.33	1.51	129.26	265.89	0.24	0.90	3.55	0.06
Peer smoking				0.41	0.90	0.21	47.81	180.98	0.07	4.74	2.42	3.83*
Self-efficacy				0.10	0.12	0.67	89.67	21.85	16.84***	−0.45	0.28	2.49
Smoking years				0.42	0.13	11.25**	44.21	25.24	3.07	−0.24	0.34	0.52

FEV1: forced expiratory volume in the first second; *P < 0.05, **P < 0.01, ***P < 0.001.
